# Assessment of Music Experiences in Navigating Depression (AMEND) through a Tour of the Room assessment model

**DOI:** 10.3389/fpsyt.2026.1700027

**Published:** 2026-02-17

**Authors:** Joanne Loewy, DeWayne Williams, Harrison Appelt, Chris Pizzute, Ingrid Wheatley-Rebling, Michal Meltzer, Julian F. Thayer

**Affiliations:** 1 Mount Sinai Health System, New York, NY, United States; 2 Icahn School of Medicine at Mount Sinai, New York, NY, United States; 3 University of California, Irvine, Irvine, CA, United States; 4 Hospital of Special Surgery, New York, NY, United States; 5 University of California Irvine School of Medicine, Irvine, CA, United States

**Keywords:** depression, music assessment, children, teens, college students, post NICU parents

## Abstract

A music psychotherapy-based assessment protocol was implemented to guide the treatment of vulnerable participants experiencing depression. Through the AMEND Lab, we created opportunities to support at-risk populations using relationship-centered music therapy. The current study examined whether such therapy delivered over a three-month period could improve well-being, as indexed by self-reported depressive symptoms and resilience. Our sample (n = 72, 50 women, mean age = 38 years, SD = 24 years) included children, teenagers and college students, adults whose neonates were recently in intensive care, and adults with mild cognitive impairment. Results indicated that individuals in the treatment group demonstrated decreases in depression and increases in resilience, whereas the control group showed no such improvements. These findings suggest that culturally relevant psycho-emotional characteristics expressed through live music therapy assessment may provide clinicians with an important resource when working with individuals who experience depression and diminished resilience. We hope these methods will strengthen future efforts to identify, recognize, and understand the ways in which music can support the assessment of resources which influence well-being across diverse populations. Such approaches may offer health professionals ways to integrate music as an accessible, social, cost-effective, and complementary assessment tool that will lead toward enhanced identification of issues that provide for a variety of treatment options.

## Introduction

The lack of person-centered resources for vulnerable individuals who face depression is not attributed to an identified population but rather to people of all ages, backgrounds and diagnoses. One problem in community health is a shortage of resources, and particularly missed opportunities for continuity of care from hospitalization to home. Furthermore, there have been few clinical trials that focus on the assessment of live, music-based strategies that reflect validity for the treatment of depression in those most vulnerable. To rectify this, our center has worked with clinical research teams across a multitude of disease trajectories for over three decades, including treatments to those most vulnerable. Our keen interest in understanding how music modalities provide a host of viable options for depression led to the development of a dedicated music psychotherapy assessment lab, whereby practicing music therapists and researchers in both hospital-based and clinical out-patient settings would understand how to consider live music as a meaningful treatment modality.

### Review of music therapy assessments

Music therapy assessments have traditionally focused on outcomes that are not necessarily directly tied to musical engagement, particularly within educational and healthcare contexts. When the goal is to examine communication skills—such as speech or language—researchers often turn to instruments grounded in cognitive or communication frameworks. For example, in cases involving movement disorders, neurologic music therapy assessments have proven effective in evaluating rhythmic movement (1). For a broader view of overall functioning, the Individualized Music Therapy Assessment Profile (2) offers a detailed and comprehensive tool for assessing both musical and non-musical abilities, highlighting a client’s strengths and areas for growth.

A growing body of music therapy assessments evaluate how musical engagement reflects communication, social functioning, and interpersonal behavior. For example, the Nordoff-Robbins assessment scales (3) examine how musical interaction reveals functional communication skills and patterns of relating to others. Similarly, the Individual Music-Centered Assessment Profile for Neurodevelopmental Disorders assesses functional capacities within a coactive music therapy relationship. Grounded in detailed musical observation, this focuses on a child’s engagement through instrument play, vocalization, movement, and social-emotional behaviors such as gesture use, eye contact, and joint attention (4).

Additional assessment tools include: (i) the Special Education Music Therapy Assessment Process (5); (ii) the Music Therapy Assessment Tool for Awareness in Disorders of Consciousness (6); and (iii) the Residual Music Skills Test (7). The latter is commonly used with individuals with Alzheimer’s disease or other neurocognitive disorders to evaluate both active musical production and receptive musical skills that tend to remain preserved despite cognitive decline—for example, singing familiar songs with and without cues, identifying instruments by sound, recalling tonal patterns, and completing rhythm tasks. The Music-based Autism Diagnostics (8) supports the identification of autism spectrum disorder in individuals with intellectual disabilities or limited verbal communication, while the Music in Dementia Assessment Scales provides a structured means of assessing musical engagement among older adults with memory impairment. The Music Therapy Assessment of Emotionally Disturbed Children (9) facilitates the evaluation of children’s functioning in and outside of music, with attention to relevant family dynamics. Many of these assessment tools incorporate psychometric indicators to strengthen the reliability, validity, and functional relevance of the derived clinical information.

In 2015, an International Music Therapy Assessment Consortium was founded “to facilitate and support the development and standardization of robust and research-based music therapy assessment tools” and to “promote the implementation of music therapy assessment in clinical practice” (10; https://www.musictherapy.aau.dk/imtac/). This created a centralized repository where clinicians, students, and researchers could submit music therapy assessment tools to build a comprehensive catalogue of assessment strategies for use in research and clinical practice. During this period, and in the years prior, our team began studying depression among individuals who experienced repeated or sudden hospitalizations – populations at heightened risk for post-hospitalization depressive symptoms. More recently, we have expanded this work to develop approaches within music psychotherapy aimed at assessing “normal neurotics,” particularly in the post-COVID context where individuals face increased emotional triggers unrelated to pulmonary functioning yet associated with persistent depressive experiences (11).

Our research has been deeply informed by Erkkilä et al.’s (12) seminal study, which combined clinical improvisation with standardized assessments to evaluate changes in depressive markers during music making. Notably, few existing assessment tools within music therapy explicitly measure psychotherapeutic processes or markers of psychological change as they unfold musically. Moreover, the commonly used descriptors “active” and “passive” music engagement are problematic. So-called passive music listening can be highly activating, whereas active music making may not elicit emotional engagement. Thus, the active/passive distinction oversimplifies the complex experiential and affective processes involved in musical engagement (13).

In the current study, we incorporated non-musical metrics alongside musically grounded relational processes to examine how sound associations and musical engagement illuminate persistent elements of depression. To do so, we employed our “Tour of the Room” music psychotherapy assessment tool (see Appendix A), which provides a structured method for exploring clients’ emotional, associative, and interpersonal responses through music-based interaction.

### The AMEND lab

We established the AMEND music therapy assessment lab to design and evaluate assessment protocols tailored to participants’ age, symptom profiles, and post-disease trajectories. Guided by a multidisciplinary team, we developed coordinated identification, intervention, and outcome pathways that allowed us to examine how music therapy may support wellness and resilience among targeted, at-risk populations. Central to this work was the use of controlled exposure to specific instrumental sounds within a structured assessment context, enabling us to evaluate participants’ responses to distinctive auditory stimuli and their potential therapeutic impact.

The AMEND lab explored the benefits of individualized, live music therapy experiences, supplemented by one web-based group intervention focused on parents of infants recently discharged from the neonatal intensive care unit (NICU). This latter cohort was selected because of its elevated risk for depression and psychosocial stress. Across projects, our aim was to understand how a distinct, unique music therapy assessment protocol could illuminate emotional functioning, depressive symptomology, and identify resources that influence broader markers of well-being. Our guiding research questions included:

1. How do individuals’ attractions, repulsions, and associative responses to specific sounds shape their engagement in music therapy experiences?
2. How can a music psychotherapy assessment protocol help identify depressive symptoms that influence quality of life, mood, affective range, and resilience across educational and medical settings?

### Depression as a global health concern

Depression remains a pervasive threat to global well-being. During the COVID-19 pandemic, rates of isolation-related depression rose sharply, contributing to the highest annual number of mental-health–related deaths recorded in the United States. Between August 2020 and February 2021, the percentage of adults reporting symptoms of depression increased from 36.4% to 41.5% (CDC, 2021). Reports of unmet mental health care needs rose during that period from 9.2% to 11.7% (14). Worldwide, depression contributes to more than 700,000 deaths by suicide annually and is the fourth leading cause of death among individuals aged 15–29 (15).

A meta-analysis of 29 studies including 80,879 youth revealed that 25.2% of children and adolescents experienced clinically elevated depressive symptoms during the first year of the pandemic—suggesting that one in four young people met the threshold for clinically significant depression (16). Barriers to effective treatment remain widespread and include limited access to mental health resources as well as insufficient training among health-care providers in identifying psychological vulnerability (17).

Primary care practices in the United States are similarly underprepared to manage depression. Physician surveys indicate that although many patients present with depressive symptoms, clinical encounters often prioritize chronic medical conditions instead. Strengthening quality metrics and incentivizing evidence-based depression care have been recommended to address this deficit (18, 19).

Moreover, individuals experiencing depression—particularly adolescents and young adults with adverse childhood experiences—report reluctance to seek mental health treatment. Concerns about stigma, workplace consequences, and social judgment contribute substantially to underutilization of services (20). This gap affects individuals across racial, cultural, and socioeconomic identities; depression is not confined to any particular demographic group, and continuity of care from hospitalization to home is often inconsistent.

### Vulnerable populations: NICU parents and other at-risk groups

Certain populations face uniquely elevated risks for developing depression. For example, mothers of infants admitted to the NICU experience significantly higher rates of postpartum depression—estimated between 28% and 70%—compared to mothers of healthy term infants (21). Despite the emotional burden on caregivers, clinical attention is typically directed toward the infant’s medical needs, often leaving parental psychological distress unaddressed. The nuanced roles of personality, cultural background, and spirituality in shaping medical and emotional outcomes are likewise understudied.

Music-based interventions have shown promise in neonatal contexts. Research involving culturally grounded *song of kin* interventions demonstrates music’s capacity to improve infant vital signs and support neuroplasticity, while reinforcing parental bonding (22, 23). These findings underscore the relevance of examining music therapy’s potential to address depressive symptoms and stress responses among NICU caregivers.

Depression, anxiety, and apathy are also prevalent in individuals with mild cognitive impairment (MCI), where they can serve as early indicators of potential progression to dementia (24). Multiple studies identify depression as a significant risk factor for cognitive decline and dementia onset (25, 26). Among youth and adolescents, a combination of biological disposition, temperament, cognitive vulnerabilities, family dynamics, academic pressures, shifting social environments, and increased exposure to digital and social networking demands all contribute to heightened risk for depression (27).

### The present study

In our AMEND lab, we developed a strategic phase-oriented music therapy plan that involved a design for implementing subsets of inclusion, according to age level and post disease participation. The AMEND lab shows distinct ways music defines and ultimately works to expand range of affect, building motivation and resilience capacity which is so often ambushed with depression. A strong assessment is paramount to a well-crafted treatment trajectory. Our consent form ensured that participants involved in any other type of creative arts therapy were not included in our study.

Our study sought to scientifically investigate strategic subsets of depressed individuals or people prone to or at-risk of depression through music experiences of individual, group, and blended supported contexts. In our investigation, meetings were held with our multi-disciplinary team, that included members of the Carnegie Hall Weill Music Institute and University affiliates. Through tabulation of participatory options with standardized depression and resilience measurements, we studied how our music therapy assessment might define and mitigate how our subsequent treatment interventions could alter self-reported depression and resilience over the three months.

## Methods

Eight-four subjects between the ages of 8–70 years with a clinical diagnosis of Major Depressive Disorder (MOD) who met the DSM-5 (2013) criteria for a Major Depressive Episode. Alternatively, subjects could have identified themselves, been identified by family, referred by a clinician as depressed, or had a history of depressive symptoms. This sample size is adequate, as prior work in this domain suggests an effect size of 0.36 and power analyses suggest 40 subjects per group (28).

**Figure 1:**
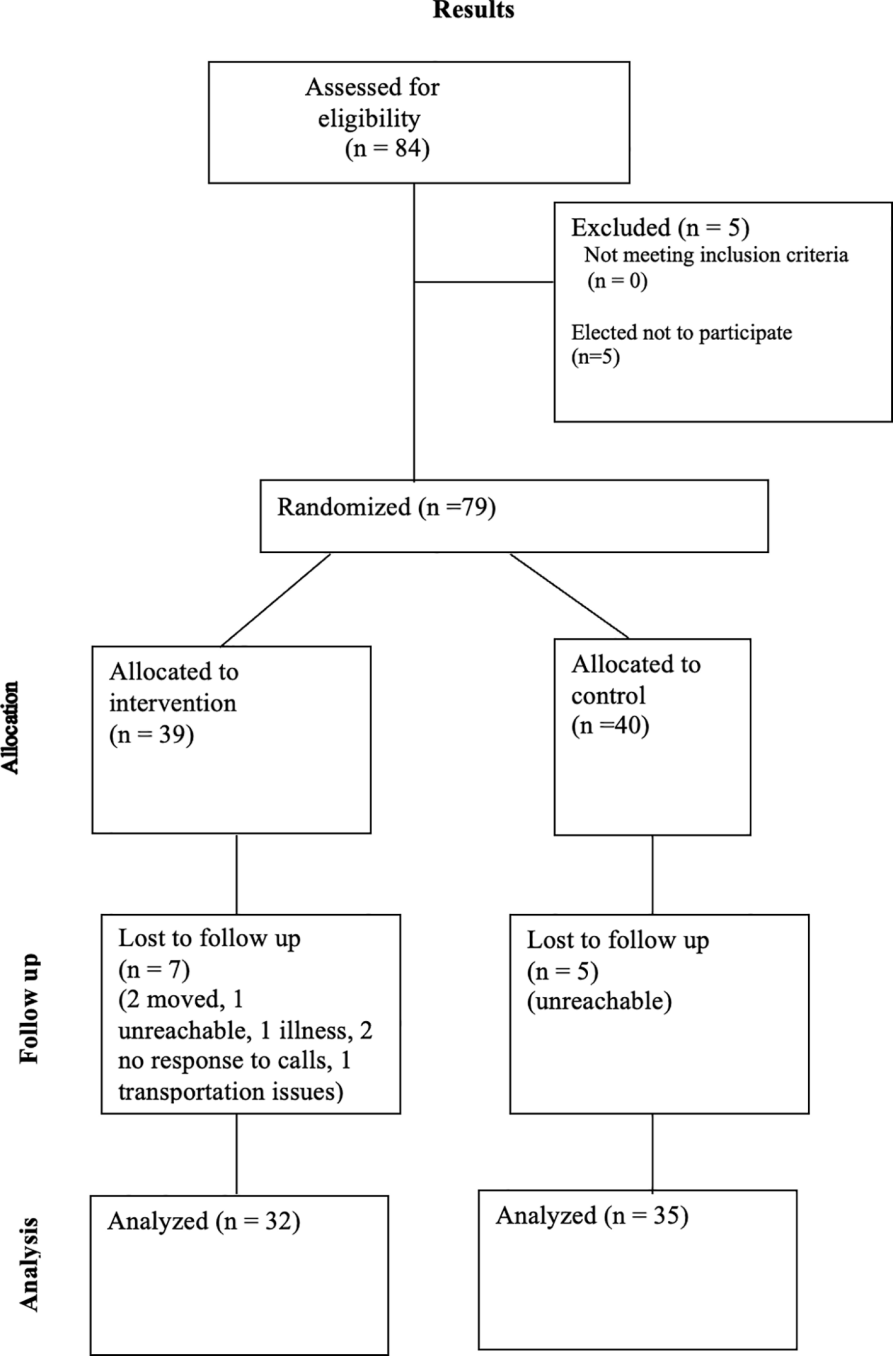
Consort flow diagram.

Inclusion criterion included (i) depressive episodes and/or observable symptoms of mild to moderate depression intensity, (ii) self-reported satisfactory bilateral hearing, and (iii) English or Spanish language fluency sufficient to complete the study. Medication status was not tabulated and remained unadjusted from usual standard of care during the study. Exclusion criterion included: (i) Axis I diagnosis, aside from MOD, considered the primary diagnosis, (ii) Bipolar Disorder Type I or II, (iii) Axis II diagnosis such as antisocial or borderline personality disorders (iv) arrhythmia, (v) pacemaker, (vi) prolapsed vertebral disc, and (vii) recent back or neck injuries (See **Figure 1** for full details).

The study was conducted in accordance with the principles of the Declaration of Helsinki and was registered with the Icahn School of Medicine IRB at Mount Sinai Hospital and registered as clinical trial #06924892.

### Procedures

Recruitment began in April with baseline evaluations overseen by a psychiatrist, psychiatric resident, or Fellow at the LACMM. The LACMM suite, located at Mount Sinai Union Square Downtown, is housed in the same building as the Neurology and Pediatric clinics, facilitating convenient referrals from these services. The site is also in close proximity to the Third Street Music School Settlement, YAI, and Cooper Union, expanding access to additional referral networks. The Mount Sinai Adolescent Health Center (MSAHC), located at 320 E 94th Street, provided a geographically accessible recruitment site near Mount Sinai Hospital. Our recruitment strategies successfully reached the vulnerable populations targeted within New York City. Across our program initiatives, systematic examination of discrete musical elements facilitated the identification of co-morbid dysthymic symptoms that compromise wellness and may serve as contributing mechanisms in the progression of neurological dysfunction.

Participants were identified through educator, clinician, family, or self-referrals, as well as through our Wellbeing Concert surveys (**Appendix B**) and on-site questionnaires. Once preliminary eligibility was established by the research team, inclusion criteria were verified and written informed consent was obtained during the initial psychiatric screening appointment.

Six music therapists participated in the study (four women and two men). All were professionally trained, board-certified (MT-BC), and held either Master’s or Doctoral degrees in music therapy; five trained in New York State and one in Israel. Throughout the study, clinicians received regular supervision, including both individual and group-based sessions, and participated in monthly meetings with a licensed supervisor experienced in both music therapy and psychotherapy. A psychiatrist was available for weekly consultation to address complex clinical or diagnostic questions.

To ensure treatment fidelity, all therapists completed extensive preparatory training prior to study implementation. This training occurred weekly over a three-month period and focused on establishing a shared understanding of the theoretical and clinical foundations of the assessment model, as well as consistency in its application. Workshops included real-time observation and practice of the “Tour of the Room” assessment procedure, allowing therapists to rehearse intervention strategies, engage in critical discussion, and receive peer and supervisory feedback in a structured seminar format.

Baseline assessments included clinician-administered measures of depressive symptom severity, using the Beck Depression Inventory (BDI) for adults and the Children’s Depression Inventory (CDI-10) for children (29). The CDI has shown evidence of strong psychometric properties, is in simple language for children, and a useful tool in assessing treatment efficacy.

Resilience was evaluated for all participants using the Connor-Davidson Resilience Scale (CD-RISC-10). Additional demographic and clinical information were collected, including self-rated symptom severity, sleep quality, quality of life, anhedonia, and cultural factors related to music use and musical history, consistent with standard practices in music therapy evaluation.

### Intervention: tour of the room

Assessment procedures included a comprehensive psychiatric evaluation to confirm each participant’s primary diagnosis, which incorporated patient, family, and/or referring physician’s reports, medical history, concomitant medications, and demographic information. Baseline assessments were completed within seven days of enrollment and prior to assignment to the music therapy or control condition. Randomization was performed by a blinded statistician who implemented a computer generated randomization list and generated a sequentially numbered randomization chart, from the computer generated.list. The music therapy intervention lasted three months and was based on the Tour of the Room (TOR) assessment model (Appendix A).

The TOR assessment, developed by the first author, is grounded in the premise that therapeutic music engagement begins with understanding how individuals experience specific sounds. Functionally, the model operates as a form of “audio Rorschach,” in which participants’ interpretations of sounds reveal underlying emotional, associative, and relational dynamics. These interpretations are encouraged and build developing clinical themes which shape clinical decision-making and help structure subsequent therapeutic intervention.

The TOR assessment begins with a curated “buffet” of 24 instruments. The music therapist samples sounds from each instrument while the participant responds with associations, memories, or affective reactions. The therapist documents these responses and after the Tour, of sampled sounds, then invites the participant to choose an instrument they are most interested in playing, or if preferred, hearing the therapist play.

Improvisation follows, shaped by the participant’s preference: the therapist may play for the participant, with the participant, or observe the participant playing independently. During this process, the therapist attends closely to improvisational style, familiarity with the instrument, requests for specific ways of playing, and relational patterns embedded in the musical interaction. Following the improvisation, reflective dialogue (e.g., “What was that like?”) helps to contextualize the participant’s musical choices and emotional experience. When time permits, both solo and dyadic (therapist–participant) improvisations are explored, providing additional diagnostic insight.

Later in the session, the therapist introduces a more personal, autobiographical component by inviting the participant to request or share a meaningful song.

This may include what Loewy (30) describes as the *song of kin*, which can be defined as a personally significant piece of music tied to a powerful memory, cultural identity, or life event. Participants may choose to sing or play this song a cappella, or with the therapist’s accompaniment, either during the assessment or in subsequent sessions. This step is intentionally placed later in the process, as it requires a greater degree of trust and personal disclosure. The individualized treatment implies that sessions were based on the TOR, and SOK themes presented through the assessment, and according to music therapy Areas of Inquiry (see **Appendix C**).

Following this initial assessment, participants completed twelve 45-minute music therapy sessions over three months each week. All sessions were video recorded, transcribed, and are currently undergoing hermeneutic analysis (data forthcoming and therefore not presented in this report). Post-intervention assessments were conducted within 1–7 days of the final session. For participants in the control group, the post-assessment occurred at the three-month mark corresponding to the treatment timeline.

Therapists’ responses to the TOR assessment adhered to an open format protocol (see **Appendix D**), informed by issues identified through the assessment including the song of kin. Themes derived from sound associations served as foundational material for therapeutic intervention and were integrated according to each participant’s level of readiness. Instances of resistance to particular themes were documented and deferred for subsequent sessions beyond the initial twelve. In some cases, issues were addressed metaphorically through musical engagement, a common practice particularly with pediatric populations, and later revisited during verbal processing. Music responsiveness constitutes a core component of therapist training, enabling targeted engagement with emergent themes. Therapeutic responses were guided by participants’ histories and the thematic material generated during sessions, with careful attention to transference and countertransference dynamics occurring in live music making contexts. **Appendix C** delineates common areas of inquiry and qualitative strategies for goal attainment. The TOR and *song of kin* frameworks are integral to follow up procedures, and the inclusion of case examples and typical associations illustrates the role of these assessments in informing treatment planning. **Appendix D** provides the template for individualized session logs inclusive of future planning.

#### Primary outcome measures

Utilizing the same scales as outlined above, follow-up measures of both depression and resilience were assessed. Our primary interest here was to assess how depression and resilience might change pre-to-post intervention.

#### Vulnerable cohorts

Several vulnerable cohorts were included in this study. Specifically, we enrolled four cohorts: children (aged 7 to 14), adolescents (aged 15 to 18), college students (aged 18 to 32) post-NICU parents, and adults aged 50 and older with Mild Cognitive Impairment (MCI). Many adolescent and MCI participants were referred directly from the hospital; either from the emergency department following suicide risk evaluations, from referring physicians in the case of MCI, or from social workers for post-NICU parents. Additional participants were recruited through local schools and colleges. We particularly aimed to support individuals navigating issues related to gender, diversity, human rights, dignity, and personal agency.

In response to the rising rates of depression among adolescents, our music therapy program at the Mount Sinai Adolescent Health Center (MSAHC) emphasized accessibility, self-expression, and self-esteem in all outreach materials (**Appendix B**). These materials were tailored for youth referred by family members, educators, or health professionals—especially those for whom prior verbal therapies had yielded limited benefit.

Participants randomized into the control group were placed on a waitlist to ensure they had access to services after the three-month study period. Their music therapy treatment data was not included in the study. Parents discharged from the NICUs seek developing relationships with their infants, which may be compromised when hospitalized, as bonding opportunities are limited. Our partner Carnegie Hall Weill Music Institute’s Lullaby Project and Big Note Little Note has enhanced bonding with young children and families for many years. (31) Our teams of neonatologists and music therapists within Mount Sinai Health system’s NICUs on the East and West sides of NYC enrolled, pre-tested and referred our in-hospital high-risk parents to Carnegie Hall Weill Music Institute for home music participation upon discharge in groups via web. Once referred by our social workers and music therapists post-NICU discharge, Carnegie musician teaching artists invited parents to participate in the Big Note Little Note sessions. The sessions illuminated the ways in which music’s potency addressed their symptoms as continuity of care from hospital to home.

It is striking how often music therapists offer instruments and sounds with only limited knowledge of their clients’ personal associations of these sounds and of former music associations. Over the years, we have witnessed profoundly meaningful outcomes from spontaneous instrument choices, but we have also seen less helpful or even counter therapeutic responses in cases where we did not have an opportunity to check in with clients about what the sounds represented to them. Below are common examples of associations expressed through Tours of the Room. These examples are not specific to the present case and were compiled through years of clinical practice:

- Piano: Past pressured childhood lessons, feelings of failure (“I suck at this,” “I do not remember anything”).
- Ocean drum: Tears or grief, one client associated it with a sibling drowning (“I cannot stop this feeling of sadness, crying”).
- Drums: Firing in combat, overwhelming intensity (“It is too much”), early emergence of anger or control themes.
- Harp: “Death,” “angels,” loss of a friend.
- Gong: “India,” “prayer,” death.
- Bells: “Salvation Army asking me for money at the subway stop,” winter holidays, loneliness.

Some of these associations have led to productive therapeutic experiences and deeper connections, and the assessment can help uncover unresolved conflicts or themes that may need to be approached with care, or deferred until the appropriate time. Our assessment functions as a type of audio Rorschach that guides intentional musical choices and reveals what may be most meaningful to clients at the beginning of therapy.

Please see **Appendix E** for TOR case study.

Knowing from the Tour that Jamie (**Appendix E**) associated the ocean drum with family trips allowed for a deeper conversation about what happens during Christmas, which became clinically relevant since Jamie’s cutting began shortly after the holiday break. Discovering that the piano evoked both failure and nurturance added additional layers of understanding about their lived experience. These small pieces of the Tour of the Room assessment offer a snapshot of how sounds can deepen our understanding of a person and provide direction for subsequent clinical work.

### Statistical analyses

Statistical tests were conducted using IBM SPSS Statistics (ver. 27, IBM Chicago, IL, USA), Stat Soft Statistica 6.0 (StatSoft, Inc., Tulsa, OK), and R (ver. 4.5.1, R Foundation for Statistical Computing, Vienna, Austria) with the packages afex, emmeans, readxl, dplyr, tidyr, and openxlsx. An intent-to-treat analysis was not performed on these data. A final sample of 75 individuals with all demographic data were included for analysis.

Means and standard deviations presented are raw (untransformed) values. Independent samples t-tests were used to determine differences between the treatment and control group on all variables of interest. Repeated measures ANOVA tests were used to determine differences in pre- and post-metrics as a function of treatment or control group (**Appendix F**). Specifically, time (pre and post) was used as the intra-subject factor. Group (treatment vs. control) was used as the inter-subject factor. Follow-up tests adjust for sex, age, and sub-groups which were dummy coded (1 = children; 2 = teenagers & college students; 3 = NICU parents; 4 = older MCI patients). We used preplanned contrasts (32) to evaluate differences in treatment effect over time between control and treatment groups.

Correlation analyses were used to determine the relationships between depression and resilience at both time points. Fisher’s r-to-z transformation was used to examine potential differences in correlations between the control and treatment groups.

All tests were two- tailed with significance set at alpha of.05. Eta (r) are presented and interpreted, as it reflects the effect size of each test. 95% confidence intervals are also presented for mean differences Line graphs are presented to display the average change trajectories for both depression and resilience from pre- to post-treatment.

## Results

Descriptive statistics for the full sample and stratified by treatment group for all variables of interest are presented in **Table 1**. Independent samples t-test showed that the treatment group had lower depression scores (t (65) = 2.60, r = .306, [0.49, 3.80], p = .012) and higher resilience scores (t (66) = -2.15, [-8.07, -0.30], r = .256, p = .035) relative to control group in the post-treatment condition only. No other significant differences for age, baseline depression, or baseline resilience emerged (each p >.615).

**Table 1A:** Sample demographics.

Sex	Full sample		Control		Treatment		CI_LL_	CI_UL_	*p*
	50 (25)		24 (12)		26 (13)		-.23	.23	.999
	M	SD	M	SD	M	SD			
Age	38.24	24.15	38.84	22.43	37.71	25.87	-10.1	12.3	.843
Pre-Depression	5.06	3.67	4.97	3.95	5.14	3.42	-1.88	1.54	.846
Post-Depression	4.00	3.45	5.11	3.82	2.89	2.66	0.70	3.74	**.005**
Pre-Resilience	26.44	6.97	26.03	7.05	26.86	6.95	-4.06	2.40	.615
Post-Resilience	28.32	7.80	25.86	8.09	30.78	6.75	-8.36	-1.48	**.007**

This table displays number of men and women per respective group (men in brackets), in addition to means (M) and standard deviations (SD) for all continuous variables of interest, including age (in years), self-reported depression, and self-reported resilience (see Methods for details). Note that for 4 participants that enrolled in the study, sex was not recorded–hence a total of 75 of the 79 participants are included in the sex statistics noted above. Reported data is sourced from the 67 of 79 participants who complete both pre- and post- surveys. For both depression and resilience, measures were taken prior to the intervention (pre-) and three months later (post-). 95% Confidence intervals (CI) are displayed, including lower (LL) and upper (UL) limits. Bolded p-values represent significant differences between treatment and control groups on respective variables (p <.05).

Repeated measures ANOVA showed a main effect of time (F (1, 65) = 5.03, [-0.05,.408], r = .268, p = .028) on depression scores such that lower depression scores were observed post-intervention. Results also showed a time by group interaction to predict depression (F (1, 65) = 5.67, r = .283, [.046, 0.49], p = .020).

**Figure 2:**
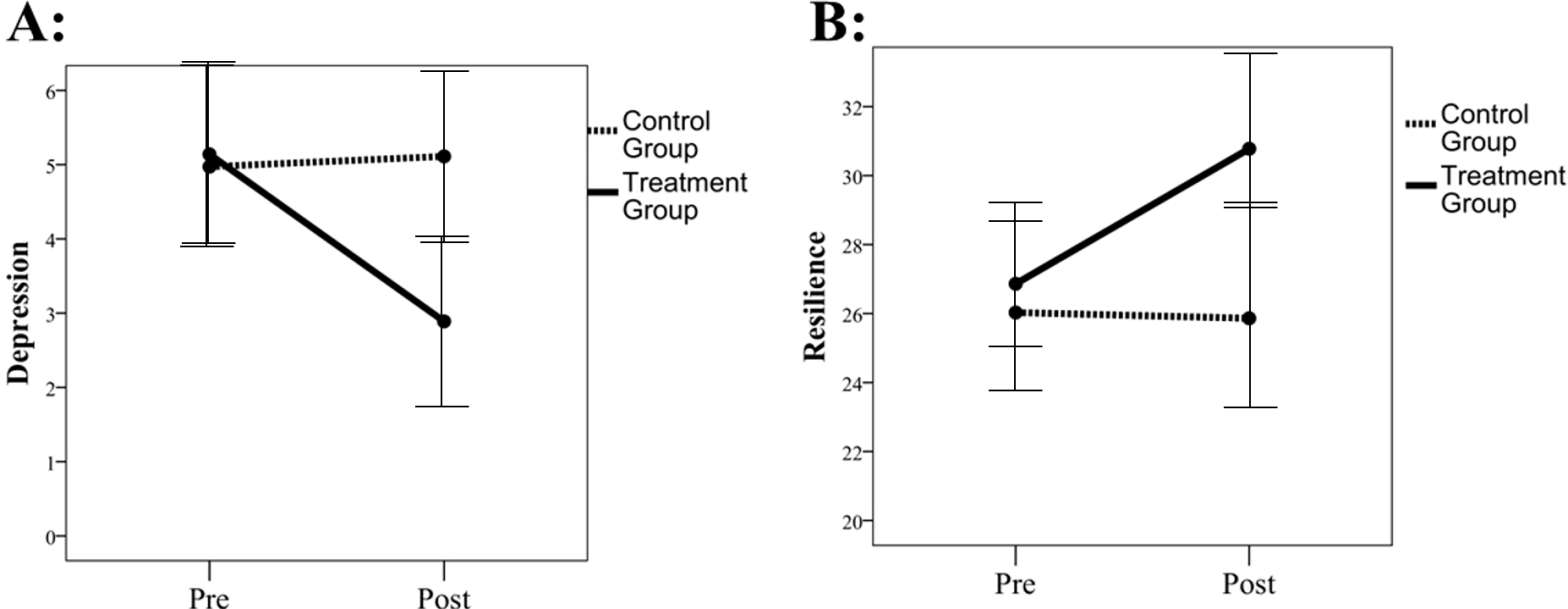
Self-reported depression and resilience over time. **(A)** represents self-reported depression from pre-intervention to post-intervention in the full sample. **(B)** represents self-reported resilience from pre-intervention to post-intervention in the full sample. Control group is represented by the dotted line, with the treatment group represented as a solid line. Error bars report 95% confidence intervals around the mean.

Preplanned contrasts showed that for the treatment group (F (1, 65) = 10.23, r = .369, [0.14, 0.56], p = 0.002), but not the control group (F (1, 65) = 0.01, r = .0.012, [-0.23, 0.25], p = .920), depression scores decreased significantly pre-to-post treatment. Adjusting for age, sex, and demographic status, results remained identical (F (1, 59) = 9.36, r = .343, [0.12, 0.59], p = .003), in that the treatment group showed decreased depression over time relative to the control group (**Figure 2A**).

Repeated measures ANOVA showed a main effect of time (F (1, 65) = 5.05, r = .267, [0.03, 0.47], [0.04, 0.47], p = .028) on resilience scores such that higher resilience scores were observed post-intervention. Results also showed a time by group interaction to predict resilience (F (1, 65) = 2.77, r = .201, [-0.04, 0.42], p = .010). Preplanned contrasts showed that for the treatment group (F (1, 66) = 7.43, r = .318, [0.08, 0.517], p = .008), but not control group (F (1, 66) = 0.17, r = .051, [-0.19, 0.29], p =0.677), resilience scores increased significantly pre-to-post treatment. Adjusting for age, sex, and demographic status, results remained identical (F (1, 59) = 6.90, r = .323, [0.99, 0.52] p = .011), in that the treatment group showed increased resilience over time relative to the control group (**Figure 2B**). Within-cohort patterns generally track the full-sample direction, although subgroup samplesizes are limited; therefore, these stratified comparisons are reported descriptively (**Table 1B**).

**Table 1B:** Stratified effects.

Cohort	Group	N	Pre-depression		Post-depression		Δ Dep (Post–pre)	Pre-resilience		Post-resilience		Δ Res (Post–pre)
			Mean	SD	Mean	SD		Mean	SD	Mean	SD	
Child (8–13) Child (8–13)	Control	4	6.75	4.43	5.50	1.91	-1.25	20.25	2.75	20.75	6.70	0.50
	Treatment	6	5.50	1.52	4.33	3.88	-1.17	22.83	9.52	26.83	5.78	4.00
Older adults with MCI Older adults with MCI	Control	10	4.70	3.59	4.60	2.80	-0.10	23.70	7.23	27.90	6.71	4.20
	Treatment	8	5.62	3.25	2.88	2.85	-2.75	28.38	6.76	32.00	5.50	3.62
Post-NICU Parents Post-NICU Parents	Control	12	3.25	2.05	4.33	4.12	1.08	31.67	4.83	29.58	10.59	-2.08
	Treatment	13	2.92	2.18	2.15	2.03	-0.77	30.69	4.57	33.00	7.42	2.31
Teens/College (14–27) Teens/College (14–27)	Control	9	7.11	5.33	6.56	5.15	-0.56	23.89	6.86	23.78	8.79	-0.11
	Treatment	5	8.00	5.00	3.60	2.70	-4.40	23.00	4.53	26.80	7.26	3.80

Cohort-stratified pre- and post-intervention depression and resilience scores by study group (means and SDs), with mean change (Δ = Post–Pre).

Results for both depression and resilience remain identical when adjusting for baseline music listening tendencies and prior exposure to music-making (each p <.05).

Zero-order correlations showed all associations between both depression and resilience at both time points to be significant in the full sample (see **Table 2A** p <.05). When stratified by control and treatment groups, the control group also showedstrong associations between all variables (see **Table 2B** p <.05). However, in the treatment group (see **Table 2C**), several associations were attenuated, including the association between pre- and post-depression (r = .297, [-0.04, 0.57], p = .079), pre-resilience and post-depression (r = -.146, [-0.45, 0.19], p = .395), and pre-depression and post-resilience (r = -.238, [-0.53, 0.10], p = .161) relationships.

**Table 2:** Correlations between variables of interest.

A: Full sample	1	2	3	4
1. Pre-depression	–	-.513^**^	.499^**^	-.280^*^
2. Pre-resilience	-.513^**^	–	-.361^**^	.557^**^
3. Post-depression	.499^**^	-.361^**^	–	-.694^**^
4. Post-resilience	-.280^*^	.557^**^	-.694^**^	–

B: Control group	1	2	3	4
1. Pre-depression	–	-.576^**^	.684^**^	-.351^*^
2. Pre-resilience	-.576^**^	–	-.519^**^	.620^**^
3. Post-depression	.684^**^	-.519^**^	–	-.658^**^
4. Post-resilience	-.351^*^	.620^**^	-.658^**^	–

C: Treatment group	1	2	3	4
1. Pre-depression	–	-.448^**^	.297	-.238
2. Pre-resilience	-.448^**^	–	-.146	.509^**^
3. Post-depression	.297	-.146	–	-.667^**^
4. Post-resilience	-.238	.509^**^	-.667^**^	–

This table displays correlations in the full sample (A) and stratified by group (B: control group; C: treatment group). Gray shaded cells represent significant differences in correlation coefficients between groups. *p <.05 **p<.01.

Comparison analyses showed that the pre- and post-depression effect differed significantly between the control and treatment groups (z = 2.15, p = .031). Although not statistically significant, a non-trivial difference was observed in the pre-resilience and post-depression comparison between groups (z = 2.15, p = .082). There was no meaningful difference between groups in the link between pre-depression and post-resilience (z = 0.5, p = .617).

## Discussion

In sum, our music psychotherapy assessment produced meaningful themes which led to treatment efficacy that indicated reductions in depression and increases in resilience over a three-month period. These effects, which were moderately strong, did not emerge in the control group, and remained robust after adjusting for subgroups (children, teenagers and college students, NICU parents, and older adults with MCI). Our findings complement recent work by Heck and colleagues (33), which demonstrated that active music engagement fosters resilience among healthy individuals and that resilience is particularly salient among depressed participants as they navigate and cope with their symptoms.

Notably, pre-treatment depression was not linked with post-treatment depression among participants receiving the intervention, whereas this link was strong and significant among the control group. This pre-to-post depression link attenuation was statistically significant, underscoring the potential of our assessment to disrupt depressive trajectories over time. Psychotherapists have emphasized the value of incorporating social skills training into depression treatment, as such integration has been shown to enhance clinical outcomes and reduce drop-out rates among individuals with depression (34). This is especially critical when working with children, a population that might lack the developmental resources to navigate major depressive stressors at home, including parental divorce, family illness, residential instability, and emerging identity concerns. In our work, these contextual stressors were highly salient among the younger participants. We believe that these factors help explain the notable improvements observed among children who engaged in musically mediated, group-based music therapy sessions, where opportunities for cooperative interaction and shared expression may provide essential scaffolding for coping and resilience.

### Implications

Cochrane reviews have examined the efficacy of music therapy delivered alongside standard care compared to standard care alone in individuals with depression (35, 36). Although the findings provide convincing support for the addition of music therapy, the authors highlighted the need for future trials employing stronger designs and samples that include children and group-based approaches. They also noted a critical gap in the literature: the lack of attention to mechanisms of action in music therapy for depression, particularly quantifiable musical characteristics that may reflect or modulate depressive symptomatology. Integrating music into patients’ plans of care therefore requires clearer definitions, structured assessments, and intentional planning. Assessment approaches with succinct definition may improve treatment outcomes.

Our study has helped address this gap by equipping healthcare teams with a more precise understanding of how prescribed music experiences may interrupt illness progression through the activation of proactive emotional and social musical states. Through the work of the AMEND Lab, we have identified specific musical mechanisms that function as behavioral agonists that preserve the capacity for wellness processes.

In doing so, our work fills a fundamental gap that has historically overlooked the specific musical mechanisms that influence and sustain quality of life, resilience, and emotional regulation, particularly in populations experiencing cognitive decline. A clearer understanding of music’s contribution to regulating mood is critical in shaping health outcomes.

### Strengths and limitations

Using standardized Depression and Resiliency scales as well as a the Areas of Inquiry and Music Qualitative Means (37, **Appendix D**), and a music history of cultural reference/relevance, our results effectively facilitated our capacity to identify, recognize, and navigate ways in which music may be helpful to a broad spectrum of health professionals and teaching artists, as an accessible, social, cost-effective, complementary activity and/or treatment option.

A few limitations are to be noted. First the intervention lasted only three months (12 sessions). This is relatively short, and no follow-up data were collected; our outcomes represent short-term effects only. We did not yet explicate what transpired during the treatment sessions of our participants. We believe that a variety of treatment options can ensue from the initial Tour of the Room. Proposing a specific style or regimen of the therapy sessions would be confining and limit how clinicians would choose to practice. Moreover, while sex/gender was not a significant covariate in the current analyses, our sample was primarily comprised of women, despite the study being open to both men and individuals who identify as non-binary. Therefore, future research should work to explore potential differences sex and gender identification differences given recent recommendations (38). Furthermore, while participant and therapist blinding was not feasible here, this remains a limitation given the potential influence of expectancy and social desirability in self-report measures. Additionally, mean depression levels in our sample did not exceed the clinical cutoff. Future studies should examine our intervention in the context of those who might be clinically depressed or unwell. It should be noted that all participants (intervention and control groups) did not have data collected on explicit changes that occurred regarding other forms of treatment. The consent form explicitly stated that the study would not change any of their current treatments. Of note, however, none of the participants received any other creative arts therapy (Music, dance, art or drama) during the timeline of our study. This was a pre-condition for receiving our therapy. Lastly, although we provide justification for the current sample size, we did not reach 40 per group due to missing data and exclusion criterion. Therefore, future studies with larger sample sizes are encouraged. This is especially true as our limited sample size did not permit a sufficiently powered analysis to detect a sufficiently significant treatment effect within each cohort.

## Conclusion

Our study examined music and associations of sounds revealed in a music psychotherapy context as a means of navigating issues related to depression, enhancing resilience, thereby positioning musical responses as culturally grounded psycho-emotional indicators that are often absent from traditional approaches in music therapy assessment. We conducted this work across multiple vulnerable cohorts and evaluated its cumulative value.

Music frequently functions as a meaningful marker for clinicians who work with individuals experiencing depression. By incorporating structured opportunities whereby music therapy assessment, is included, providing opportunities for both music making and music listening, clinicians can more deliberately tailor interventions. Musical experiences may provide accessible pathways for emotional expression and support the natural emergence of resilience capacities that unfold organically within therapeutic encounters. We hope that future assessment trials will continue to adopt and refine this promising direction, further exploring how music experiences can inform, enhance, and ultimately strengthen the assessment and treatment of depression.

## Data Availability

The raw data supporting the conclusions of this article will be made available by the authors, without undue reservation.

## Appendix A

**Table:**
