# Supplementary material for: Assessment of Music Experiences in Navigating Depression (AMEND) through a Tour of the Room assessment model
Source: Front Psychiatry. 2026 Feb 17;17:1700027. doi: 10.3389/fpsyt.2026.1700027 (PMC12983230; doi:10.3389/fpsyt.2026.1700027)
Supplement: Supplementary file 1 [file Supplementaryfile1.pdf]

Appendix B

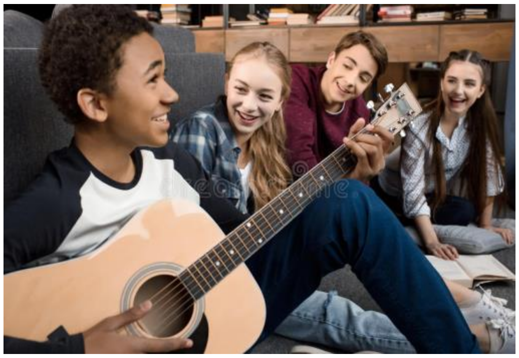

AMEND

(Assessment of Music Experiences in Navigating Depression)

Mount Sinai Health System's Louis Armstrong Center for Music and Medicine, in partnership with Carnegie Hall's Weill Music Institute, is studying how music can affect mood.

Past research indicates social-emotional benefits of music participation in individual and/or group settings, specifically for children and teens.

Participation will involve convenient set-up of a 30-minute enrollment time. Data will be collected to understand contextual factors such as participant's prior levels of experience with music.

Additional partners in this study include Cooper Union, Third Street Music School, Young Adults Institute, and Lincoln Center Moments.

MONTHLY CONCERTS WITH CARNEGIE HALL WEILL INSTITUTE

As part of this research, you may be invited to attend optional free monthly concerts at Carnegie Hall, Cooper Union College, Third Street Music Settlement or Lincoln Center. We will send you a concert schedule and will track your attendance.

Frequently Asked Questions

If I decide to participate, can I change my mind later

Yes. You can change your mind and stop being in the study at any time.

Where will the study visits be?

The visits will take place at The Louis Armstrong Center for Music and Medicine at Mount Sinai Union Square 10 Union Square East, NYC.

Our research assistants will schedule a time that works for you.

Do I need to know how to play an instrument?

No, the music experiences and discussions about music are completely voluntary and require no music training.

Appendix C- Loewy (2000), Music Therapy Assessment

Area of Inquiry	Qualitative Means
1. Awareness of self, others & of the moment	Musical, verbal, nonverbal reflection
2. Thematic Listening	Instrument, song choice, quality & style of singing and playing
3. Listening	Receptivity, ability to hear others
4. Performing	Speaking, playing, singing alone
5. Collaboration/Relationship	Willingness to interact in activity together, quality of expressing with others
6. Concentration	Ability to focus in and out of the music
7. Range of Affect	Qualities of expression, variety of moods & themes, dynamic variance
8. Investment/Motivation	Willingness to build musical experience or conversation, sustain involvement in the music al-verbal dialogue
9. Use of Structure	Reaction to space-boundaries, adherence/resistance to formatted themes/improvisation
10. Integration	How forms (music, words, feelings, songs, thoughts) are put together
11. Self Esteem	Evaluation of the created themes-taping
12. Risk Taking	Experimenting, trying something new, playing alone & together w/others
13. Independence	Ability to separate self/others musically & verbally

Appendix D: Study session notes

MOUNT SINAI HEALTH SYSTEM

THE LOUIS ARMSTRONG MUSIC THERAPY DEPARMENT

Participants: {MT PARTICIPANT #: }

Referred by:

Music Therapy Referral: {Music Therapy referral notes}

Reason for referral:

Instruments TOR associations/ Activity description:

Voice (Quality, Timbre, Pitch):

Description of the music:

Significant Issues/Theme:

Follow-up:

Acuity of Patient:

Music Therapist:

Appendix E: Case report

Case report: Jamie (a false name used to maintain privacy), born male and identifying as Jamie (age 15), was referred from the emergency room by the nurse manager. They were seen for a "Tour of the Room" assessment post discharge and subsequently included in our AMEND Lab study. Jamie was the middle child of a Hispanic family and was identified, by both their parent and themselves, as genderfluid (pronouns: they and them).

When first seen with their father, who described himself as a "stay at home Dad," the two appeared close. Jamie joked about their family, and some of the humor was clearly private. I observed without intervening. Jamie's father spoke openly about ongoing struggles at school, challenges with peers, and the family's considerable worry about Jamie's well-being. Jamie had been engaging in cutting, staying awake most of the night "on the computer," and resisting going to school. Weight loss and lack of sleep were also mentioned.

Upon entering the music room, after reviewing the study details, I noticed Jamie and their father's interest in the instruments and offered them a moment to "warm up" together. Jamie chose the bells for their father and the ocean drum for themselves, playing quietly and teasing their father in a deliberately playful, almost cunning way. I joined briefly on the piano, matching the key of the bells (F), until the random and somewhat disconnected sounds settled. "Cool," Jamie remarked. "I like these sounds together… it reminds me of visiting Aunt Sarah by the lake." "A place we go each Christmas for the holidays," added their father. "My sister's." Soon after, Dad was invited to wait in the lobby so the assessment could proceed. I then presented the "Tour of the Room" assessment to Jamie and invited their in- the- moment associations to each instrument sound.

Once all instrument associations had been identified, Jamie was invited to choose one to play, along with a single sound they would like the therapist to add. This serves as a subtle way of exploring whether they wanted accompaniment. Jamie chose the ocean drum and placed me on the guitar, explaining that their sister takes lessons. As we played, Jamie looked directly at me and asked if I could play "MacArthur Park," which they described as a favorite song learned from their aunt years earlier. When I asked whether this was the same aunt they visit for Christmas, they giggled and said "Yes," as if surprised I had made the connection. They added that their sister "knows how to play it." As I began the song on guitar, Jamie sang softly. This moment led me to wonder whether this might be Jamie's *song of kin,* especially considering their close relationship with their older sister and the emotional associations expressed during the Tour of the Room.

Appendix F. ANOVA Analysis

Outcome	Effect	Numerator_df	Denominator_df	Mean Squared Error	F	p	r	r_CI_lo	r_CI_hi
Depression	group	1	65	19.36300137	2.33876489	0.13104188	0.18636344	-0.0563686	0.40829431
Depression	time	1	65	5.681572802	5.03000945	0.02832814	0.26800464	0.02970856	0.47747485
Depression	group:time	1	65	5.681572802	5.67099235	0.02018591	0.28327545	0.04621128	0.49013288
Resilience	group	1	66	86.72013643	2.89335537	0.09365294	0.20493315	-0.0352125	0.4227044
Resilience	time	1	66	26.4918536	5.04958839	0.02798089	0.26659206	0.03007885	0.47483554
Resilience	group:time	1	66	26.4918536	2.77141858	0.10070186	0.20074608	-0.0395731	0.41911129
